# Second-line chemotherapy efficacy is not affected by prior exposure to immunotherapy in extensive-disease small-cell lung cancer: results from the ESME French national real-world cohort

**DOI:** 10.1016/j.esmoop.2026.108324

**Published:** 2026-08-07

**Authors:** E. Gobbini, M.H. Diallo, C. Clément-Duchene, C. Chouaid, S. Hiret, C. Audigier-Valette, A. Bizieux, D. Debieuvre, X. Quantin, Y. Le Guen, A. Lemoine, L. Falchero, J. Remon-Masip, L. Bosquet, M. Pérol, N. Girard

**Affiliations:** 1 Thorax Institut Curie Montsouris, Institut Curie, Paris, France; 2 Biometry Department, Institut Curie, Paris, France; 3 Medical Oncology Department, Institut de Cancérologie de Lorraine, Vandoeuvre-lès-Nancy, France; 4 Pneumology Department, CHI Créteil, Créteil, France; 5 Medical Oncology Department, Institut de Cancérologie de l’Ouest, Nantes Saint Herblain, France; 6 Pneumology Department, CH Sainte Musse, Toulon, France; 7 Centre Hospitalier Départemental, La Roche sur Yon, France; 8 Pneumology Department, Groupe Hospitalier Mulhouse Sud Alsace, Mulhouse, France; 9 Montpellier Cancer Institut (ICM) and Montpellier Cancer Research Institute (IRCM), INSERM U1194, University of Montpellier, Montpellier, France; 10 Centre Hospitalier Universitaire, Rennes, France; 11 Oncology Department, Institut Jean Godinot, Reims, France; 12 Pulmonology and Thoracic Cancer Unit, Hôpital Nord-Ouest, Villefranche-sur-Saône, France; 13 Oncology Department, Gustave Roussy, Villejuif, France; 14 Health Data & Partnership Department, Unicancer, Paris, France; 15 Department of Medical Oncology, Centre Léon Bérard, Lyon, France

**Keywords:** immunotherapy, real-world data, SCLC, second-line

## Abstract

**Background:**

Extensive-disease small-cell lung cancer (ED-SCLC) is an aggressive disease with limited effective therapeutic options. Clinical trials suggest an additive effect of chemotherapy and immunotherapy in SCLC, although the magnitude of benefit seems to be limited. Considering the prolonged peripheral persistence of anti-PD-(L)1 blockade, we wanted to investigate the impact of prior immunotherapy exposure on the efficacy of second-line chemotherapy.

**Patients and methods:**

Using the Epidemio-Strategy and Medical Economics lung cancer national real-world database, we selected ED-SCLC patients diagnosed between 17 February 2010 and 4 December 2023 who had received at least two lines of treatment for a metastatic disease with platinum-based regimens in the first-line setting. Second-line real-world progression-free survival (rw-PFS) and overall survival (OS) were analyzed according to first-line immunotherapy exposure using a multivariable Cox proportional hazards model and a propensity score-weighted sensitivity analysis.

**Results:**

The study included 2051 patients with ED-SCLC: 638 (30%) in the immunotherapy-pretreated group and 1413 (70%) who received only first-line chemotherapy. Multivariable analysis showed no significant difference in second-line median rw-PFS between immunotherapy-pretreated patients [2.9 months, 95% confidence interval (CI) 2.7-3.3] and chemotherapy-only pretreated patients (2.5 months, 95% CI 2.4-2.7) (hazard ratio 0.94, 95% CI 0.85-1.04, *P* = 0.21). Similar results were observed in the propensity score adjusted Cox regression sensitivity analysis. A longer median OS from the start of second-line treatment was observed in immunotherapy-pretreated patients (6.6 months, 95% CI 6.1-7.1 compared with 5.7 months, 95% CI 5.4-6.1, *P* = 0.03) although, this result has not been confirmed in a more contemporary cohort.

**Conclusion:**

This study showed no OS gain in second-line treatment among patients pretreated with immunotherapy compared with those who received chemotherapy alone as first-line treatment.

## Highlights

• Prior exposure to immunotherapy does not influence PFS of second-line chemotherapy in advanced SCLC.
• A longer median OS from second-line was observed in immunotherapy-pretreated patients with advanced SCLC.
• The modest absolute OS gain observed in immunotherapy-pretreated patients was not confirmed in a more contemporary cohort.

## Introduction

Small-cell lung cancer (SCLC) accounts for 13% to 15% of lung cancers and is characterized by rapid growth, early dissemination, and high initial sensitivity to platinum–etoposide chemotherapy.^1^ Despite high response rates to first-line therapy, nearly all patients relapse within months, and survival beyond 5 years remains poor in the metastatic setting.^2^ The introduction of immune checkpoint inhibitors such as atezolizumab and durvalumab in the first-line setting has only modestly improved survival but has established chemoimmunotherapy as the new standard of care in extensive-stage disease.^3^^,^^4^ Second-line chemotherapy for relapsed SCLC typically yields modest outcomes, with median overall survival (OS) ranging between 2 and 6 months, heavily influenced by performance status, disease extent, and platinum sensitivity.^5^, ^6^, ^7^ Topotecan was the only approved option until 2020, achieving response rates ranging from 8% to 27% and a median OS ranging from 3.7 to 12.5 months, mostly in platinum-sensitive patients.^8^ In 2020, lurbinectedin received accelerated Food and Drug Administration (FDA) approval and compassionate access in France based on a phase II basket trial reporting an overall response rate (ORR) of 35.2% and a median OS of 9.3 months.^9^ Other chemotherapy options include irinotecan, CAV (cyclophosphamide–adriamycine–vincristine) and rechallenge with platinum-based regimens in platinum-sensitive patients.^10^

The efficacy of second-line chemotherapy after immunotherapy exposure remains uncertain. Tracking PD-1 receptor occupancy on T cells after discontinuation of nivolumab has shown persistence of bound antibody for several months after the last dose of immunotherapy independently from how long the exposure was.^11^ Second-line treatment strategies may thus be influenced by prior exposure to immunotherapy in a synergistic manner. Indeed, chemotherapeutic agents induce DNA damage and apoptosis, leading to immunogenic cell death characterized by the release of danger-associated molecular patterns such as ATP and HMGB1, which promote dendritic cell activation and antigen presentation.^12^ For example, lurbinectedin exerts immunomodulatory effects by depleting tumor-associated macrophages (TAMs), thereby alleviating immunosuppression within the tumor microenvironment (TME), and by activating the STING pathway, which increases interferon signaling, proinflammatory chemokines, and major histocompatibility complex class I expression in SCLC models.^13^^,^^14^ Similarly, topoisomerase I inhibitors like topotecan enhance tumor antigen exposure and may upregulate PD-L1 expression on tumor cells through DNA damage-induced interferon signaling.^15^ These mechanisms provide a strong rationale for synergy between second-line chemotherapy and PD-1/PD-L1 blockade or other agents targeting the TME, as chemotherapy can elicit an immune response that is further unleashed by immunotherapy. In the non-small-cell lung cancer (NSCLC) setting, the PFS2 analyses—defined as the time from randomization in the first-line setting to progression on second-line therapy or death—from the KEYNOTE-189 and KEYNOTE-407 trials supported a sustained benefit of immunotherapy beyond first-line.^16^^,^^17^ In these trials, a longer PFS2 was achieved when chemotherapy was combined to pembrolizumab compared with chemotherapy alone even though most patients in the control arm received immunotherapy after progression. These results support the idea that initial exposure to immunotherapy favorably reshapes the TME, thereby enhancing the effectiveness of subsequent treatments, including chemotherapy. Conversely, little is known about the impact of first-line immunotherapy on second-line treatment in SCLC.

The aim of the present study is to investigate whether prior exposure to first-line immunotherapy modifies the efficacy of second-line treatments in patients with extensive-disease SCLC (ED-SCLC) in the French national real-world Epidemio-Strategy and Medical Economics (ESME) cohort.

## Patients and methods

### Study population

The ESME lung cancer database is an ongoing unique national real-world cohort (ClinicalTrials.gov NCT03848052) derived from electronic health records of consecutive patients treated for lung cancer at 38 participating sites (including private nonprofit comprehensive cancer centers and public hospitals). Data are routinely collected using a retrospective data collection process. The ESME research program received legal authorization from the French Data Protection Authority (Commission Nationale de l’Informatique et des Libertés, CNIL). In accordance with French regulations governing retrospective analyses of deidentified electronic health records, formal written informed consent from individual patients was not required. However, all patients were informed about the reuse of their electronically recorded data for research purposes and retained the right to object to its use at any time. The study protocol was approved by an independent scientific and ethics committee, and all data management and research procedures adhered strictly to the Declaration of Helsinki and General Data Protection Regulation requirements.

**Figure 1:**
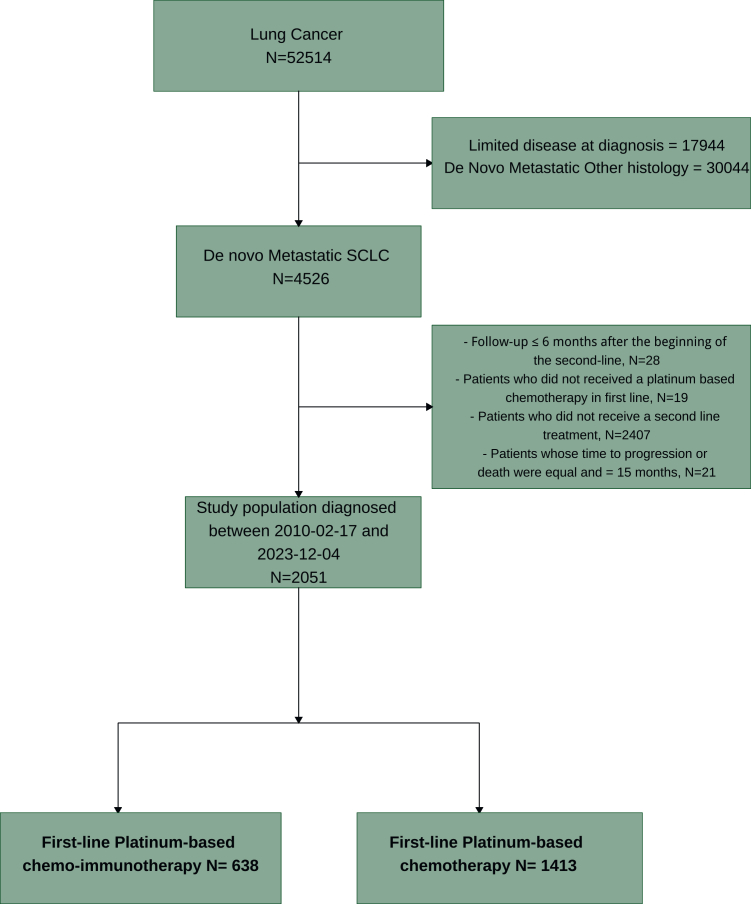
**Study flowchart.** Overview of the study design and patient selection workflow. SCLC, small-cell lung cancer.

We selected patients with *de novo* ED-SCLC diagnosed between 17 February 2010 and 4 December 2023 who had received at least two lines of treatment for metastatic disease. This long inclusion period was justified by the need to include patients who had not received immunotherapy in the first-line setting, as they were treated before the introduction of first-line chemoimmunotherapy as the standard of care (atezolizumab received approval in France in 2019, whereas durvalumab was approved in 2020). Patients had to have received a platinum-based first-line treatment and a minimum follow-up of 6 months after the initiation of the second-line treatment (Figure 1). Patients whose time to progression and death were equal and ≥15 months were considered lost to follow-up and were excluded from the study population (*n* = 21). The flowchart summarizes the selection process for identifying the target population using the ESME dataset.

### Statistical analysis

Data are summarized using sample size and percentages. Between-group comparisons for categorical variables were carried out using Pearson’s chi-square test or Fisher’s exact test (two-tailed), as appropriate. Real-world progression-free survival (rw-PFS) during second-line treatment was defined as the time from the initiation of second-line treatment to the first occurrence of disease progression, defined as the appearance of a new metastatic site, regional progression, or progression of a previously identified metastatic lesion or the end of chemotherapy due to progression, or death. Patients without events were censored at the end date of second-line treatment if available or otherwise at the date of last follow-up. OS was defined as the time from the initiation of second-line treatment to the date of death; patients alive were censored at the date of last contact. PFS and OS were analyzed as time-to-event variables using Kaplan–Meier curves, with differences between groups assessed via log-rank tests. A Cox proportional hazards model was employed and adjusted for PFS- or OS-related factors including sex, age, performance status, and the number of metastatic sites at the start of second-line treatment, smoking status at diagnosis, and platinum sensitivity (defined as a progression-free interval from first-line platinum-based chemotherapy completion ≥90 days). All explanatory variables associated with the outcome at a conservative *P* value of 0.10 (*P* < 0.1) were included in the multivariable model. To enhance the robustness of our findings, a sensitivity analysis was conducted using propensity score methods, specifically inverse probability of treatment weighting, to account for baseline imbalances between the two groups. All analyses were carried out using R software (version 4.4.1; R Foundation for Statistical Computing, Vienna, Austria), with a two-sided significance level of 5%.

## Results

### Patient characteristics

The study population comprised 2051 patients with *de novo* ED-SCLC who received first-line platinum-based chemotherapy with (638 of 2051, 30%) or without immunotherapy (1413 of 2051, 70%) and at least one second-line treatment (Figure 1). The median follow-up for the overall population was 44 months [95% confidence interval (CI) 36-68]. In patients who received first-line immunotherapy, the median follow-up was 28 months (95% CI 23-32) compared with 67 months (95% CI 61-not evaluable) for those treated with chemotherapy alone.

Significant differences between the two groups were observed for the cumulative number of metastatic sites at the start of second-line treatment. Patients treated with first-line chemoimmunotherapy had a higher proportion of ≥3 metastatic sites than did those treated with chemotherapy alone (29% compared with 21.8%; *P* < 0.001 Table 1). In addition, patients pretreated with immunotherapy more frequently had an Eastern Cooperative Oncology Group performance status (ECOG PS) of 0 to 1, both at baseline (61.9% compared with 44.9%; *P* < 0.001) and at second-line start (48.4% compared with 37.8%; *P* < 0.001). Conversely, a lower proportion of platinum-sensitive disease was observed in the immunotherapy-pretreated group compared with the chemotherapy-only group (33.4% and 39.5%, respectively; *P* < 0.001). Finally, a higher percentage of patients underwent prophylactic cerebral irradiation in the chemotherapy-only pretreated group compared with the immunotherapy group (19% and 3.4%, respectively; *P* < 0.001). No significant differences were detected in other prognostic factors, including age, smoking status and comorbidities (Table 1 and Supplementary Table S1, available at https://doi.org/10.1016/j.esmoop.2026.108324).

**Table 1:** Clinical characteristics of patients in the study population

Variable	*N*	Immunotherapy in L1		
		No	Yes	*P* value
		*N* = 1413	*N* = 638	
Sex	2051			0.19
Male		863 (61.08)	409 (64.11)	
Female		550 (38.92)	229 (35.89)	
Unknown		0	0	
Age at diagnosis, years	2051			0.10
<50		87 (6.16)	27 (4.23)	
50-60		379 (26.82)	153 (23.98)	
60-70		593 (41.97)	270 (42.32)	
70-80		308 (21.80)	166 (26.02)	
≥80		46 (3.26)	22 (3.45)	
Unknown		0	0	
Age at first diagnosis of ED-SCLC, years	2051			0.10
<50		88 (6.23)	27 (4.23)	
50-60		379 (26.82)	153 (23.98)	
60-70		590 (41.76)	270 (42.32)	
70-80		310 (21.94)	166 (26.02)	
≥80		46 (3.26)	22 (3.45)	
Unknown		0	0	
CSI^a^	1094			0.97
Other than CSI		26 (3.49)	13 (3.71)	
1 to 2		671 (90.19)	316 (90.29)	
>2		47 (6.32)	21 (6.00)	
Unknown		0	0	
Number of metastatic sites at diagnosis	2051			0.068
≤3		1364 (96.53)	605 (94.83)	
>3		49 (3.47)	33 (5.17)	
Unknown		0	0	
Cumulative number of metastatic sites at start of second-line treatment	2051			<0.001
≤3		1105 (78.20)	453 (71.00)	
>3		308 (21.80)	185 (29.00)	
Unknown		0	0	
ECOG PS at diagnosis of ED-SCLC	2051			<0.001
0-1		634 (44.87)	395 (61.91)	
≥2		264 (18.68)	105 (16.46)	
Unknown		515 (36.45)	138 (21.63)	
ECOG PS at second-line start	2051			<0.001
0-1		534 (37.79)	309 (48.43)	
≥2		342 (24.20)	154 (24.14)	
Unknown		537 (38.00)	175 (27.43)	
Prophylactic cerebral irradiation	2051			<0.001
No		1141 (81)	616 (97)	
Yes		272 (19)	22 (3.4)	
Unknown		0	0	

Values are *n* (%). Pearson’s chi-square test was used to determine the *P* value.

CSI, comorbidities of special interest; ECOG PS, Eastern Cooperative Oncology Group performance status; ED-SCLC, extensive-disease small-cell lung cancer; L1, first line.

a The comorbidities considered of special interest were chronic obstructive pulmonary disease, renal insufficiency, heart failure, diabetes, and hypertension.

### Real-world progression-free survival

**Figure 2:**
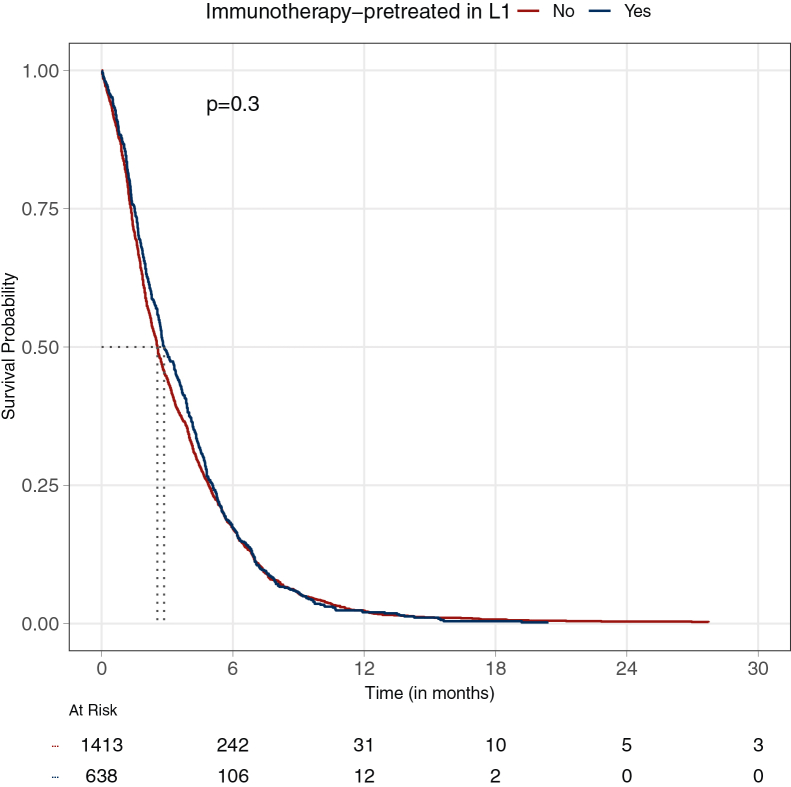
**Second-line outcomes analysis according to L1 treatment regimen.** Kaplan–Meier analysis of PFS from the beginning of second-line treatment in the study population according to the upfront treatment strategy. L1, first-line; PFS, progression-free survival.

**Figure 3:**
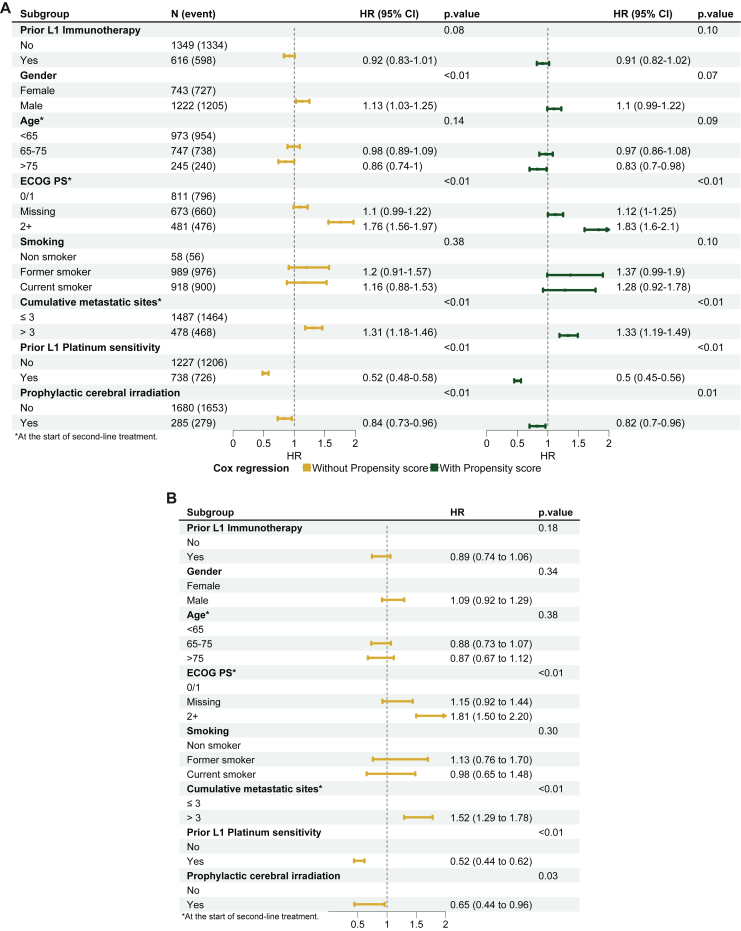
**Multivariable Cox regression analysis on second-line PFS.** (A) Multivariable Cox regression analysis of PFS in the global study population, adjusted for potential confounding factors using a propensity score (IPTW method). (B) Multivariable Cox regression analysis of PFS in the contemporary population, adjusted for potential confounding factors using IPTW. CI, confidence interval; ECOG PS, Eastern Cooperative Oncology Group performance status; HR, hazard ratio; IPTW, inverse probability of treatment weighting; L1, first-line; PFS, progression-free survival.

As per selection criteria, all patients received a second-line treatment. The most commonly used regimen was platinum–etoposide rechallenge, followed by topotecan–irinotecan, lurbinectedin, and CAV, accounting for 31%, 24%, 9.2%, and 8.9% of treatments, respectively (Supplementary Table S2, available at https://doi.org/10.1016/j.esmoop.2026.108324). The median rw-PFS in the second-line setting, irrespective of treatment type, was comparable between patients previously exposed to immunotherapy and those who were not: 2.9 months (95% CI 2.7-3.3) and 2.5 months (95% CI 2.4-2.7), respectively (Figure 2). Similar results were observed across second-line treatment options, with the exception of topotecan–irinotecan, for which patients who had not received first-line immunotherapy achieved a slightly longer, though not clinically meaningful, rw-PFS [1.7 months (95% CI 1.5-2.2) compared with 2.0 months (95% CI 1.9-2.2)] (Supplementary Figure S1, available at https://doi.org/10.1016/j.esmoop.2026.108324). Univariable Cox regression for prognostic factors likely to influence rw-PFS showed poorer outcome in male patients, those with ≥3 metastatic sites at the start of second-line treatment, platinum-resistant disease, and an ECOG PS ≥2 (Supplementary Table S3, available at https://doi.org/10.1016/j.esmoop.2026.108324). A multivariable analysis carried out with and without propensity score adjustment, and including these clinically relevant factors, was then conducted to assess the independent effect of first-line immunotherapy on the second-line treatment outcomes. Overall, no significant difference in second-line rw-PFS was observed between patients who received first-line immunotherapy and those who did not, even after controlling for potential confounding factors using propensity score adjustment [hazard ratio (HR) 0.94, 95% CI 0.85-1.04, *P* = 0.21] (Figure 3A). These results were consistent across all second-line treatment subgroups, with the exception of topotecan–irinotecan, for which improved rw-PFS was confirmed for patients who had not received first-line immunotherapy after propensity score adjustment (Supplementary Figure S2, available at https://doi.org/10.1016/j.esmoop.2026.108324). Given the modification of standard of care in terms of imaging, radiotherapy protocols, and supportive care during the study period, we tested a more recent population by selecting patients with a diagnosis after 1 January 2019 (when immunotherapy became available in France). This selection reduced the sample size of the immunotherapy-pretreated group to *N* = 615 and that of the chemotherapy-pretreated group to *N* = 274. A propensity score-weighted multivariate analysis was run on this contemporary cohort, confirming the lack of impact of first-line immunotherapy on second-line PFS (Figure 3B).

### Overall survival

**Figure 4:**
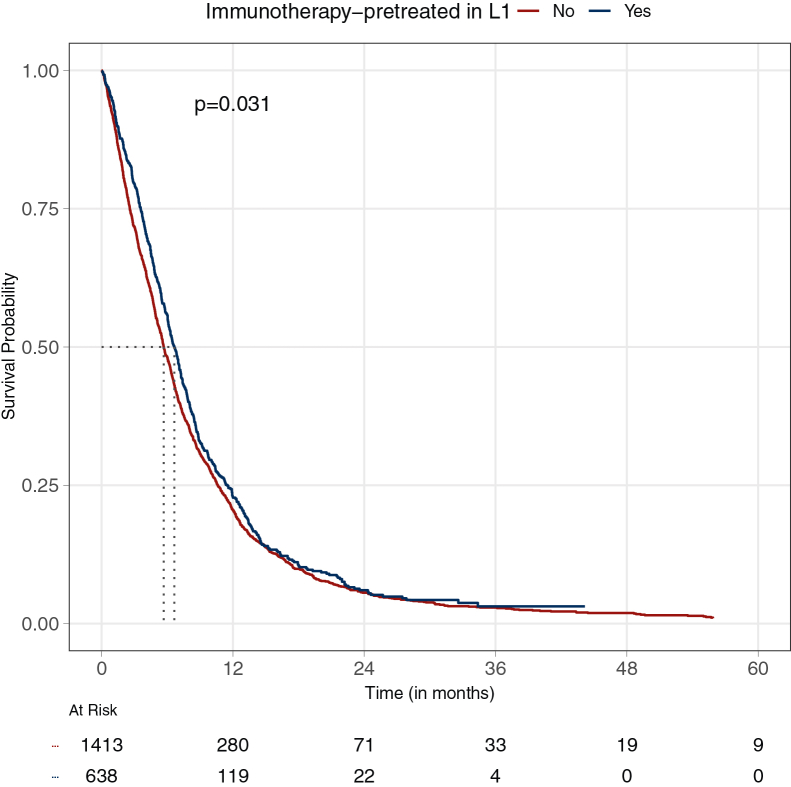
**Second-line outcomes analysis according to L1 treatment regimen.** Kaplan–Meier analysis of OS from the beginning of second-line treatment in the study population according to the upfront treatment strategy. L1, first-line; OS, overall survival.

**Figure 5:**
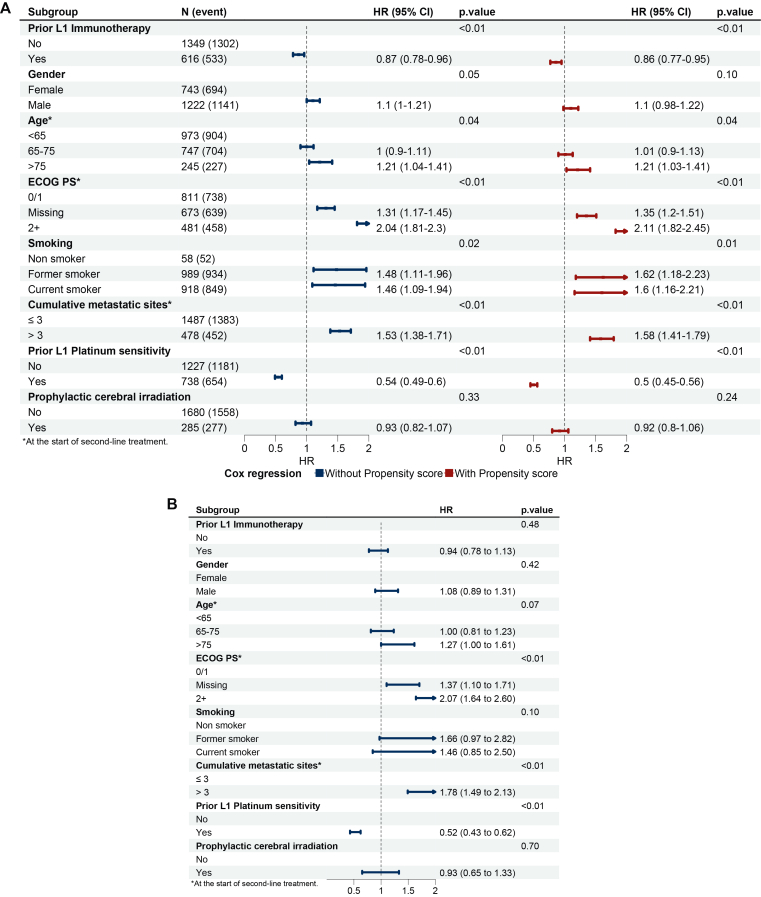
**Multivariable Cox regression analysis on second-line OS.** (A) Multivariable Cox regression analysis of OS in the global study population, adjusted for potential confounding factors using a propensity score (IPTW method). (B) Multivariable Cox regression analysis of OS in the contemporary population, adjusted for potential confounding factors using a propensity score (IPTW method). CI, confidence interval; ECOG PS, Eastern Cooperative Oncology Group performance status; HR, hazard ratio; IPTW, inverse probability of treatment weighting; L1, first-line; OS; overall survival.

The median OS from the start of second-line treatment was 6.6 months (95% CI 6.1-7.1) in the group of patients who received first-line immunotherapy and 5.7 months (95% CI 5.4-6.1) in those who did not (Figure 4). The log-rank test demonstrated a statistically significant, although not clinically meaningful, difference between the two groups in favor of patients who received first-line immunotherapy (*P* = 0.031). Multivariable analysis confirmed a second-line OS benefit for patients pretreated with immunotherapy (HR 0.88, 95% CI 0.79-0.97, *P* = 0.012) after adjustment for potential confounding factors (Figure 5A). This finding was further supported by propensity score analysis (HR 0.87, 95% CI 0.78-0.96, *P* = 0.008). However, the second-line OS benefit for immunotherapy-pretreated patients was no longer observed in the contemporary cohort (Figure 5B). Finally, no specific therapeutic strategy could be identified to account for this benefit, as OS advantage in immunotherapy-pretreated patients was observed when analyzing each type of second-line treatment separately (Supplementary Figure S3, available at https://doi.org/10.1016/j.esmoop.2026.108324).

## Discussion

The therapeutic landscape of SCLC is rapidly evolving, with new generation agents raising the bar along with new sequential treatment strategies. Based on preclinical data suggesting a potential synergistic effect between chemotherapy agents commonly used in the second-line setting and PD-1/PD-L1 inhibitors, two prospective clinical trials have recently investigated the benefit of such combination.^18^^,^^19^ The phase I-II 2SMALL trial evaluated the efficacy of lurbinectedin plus atezolizumab in relapsed SCLC.^19^ The combination achieved an ORR of 37.4%, a median PFS of 4.4 months, and a median OS of 9.5 months in patients previously exposed to immunotherapy (cohort 2), with slightly better outcomes in platinum-sensitive patients. In patients without prior immunotherapy exposure (cohort 1), the ORR was 44.1%, with a median PFS of 4.9 months and median OS of 11 months. Similarly, in the LUPER phase I-II study, patients who did not receive first-line immunotherapy achieved an ORR of 46.4%, a median PFS of 4.6 months, and a median OS of 10.5 months.^20^ Overall, these results were similar to those previously reported with lurbinectedin monotherapy, suggesting a limited contribution from immunotherapy.^9^ Conversely, the phase III IMforte trial, which compared lurbinectedin plus atezolizumab maintenance versus atezolizumab alone after chemoimmunotherapy induction, demonstrated improved median PFS (5.4 compared with 2.1 months) and OS (13.2 compared with 10.6 months) in favor of the combination.^18^ Nonetheless, whether these results can be attributed solely to the anticipation of second-line treatment remains unclear, as only 16.6% (22 of 132) of patients undergoing subsequent anticancer treatments in the control arm received lurbinectedin. In our study, prior exposure to immunotherapy was associated with a small but statistically significant OS benefit in the second-line setting. However, no specific treatment strategy appears to drive these outcomes, likely due to limited sample sizes in each group. Importantly, the modest absolute OS gain (0.9 months) suggests a limited synergistic effect between chemotherapy and immunotherapy in SCLC compared with NSCLC, as reported in pivotal clinical trials.^2^^,^^4^^,^^16^^,^^17^ Importantly, this clinical benefit was not confirmed when restricting the analysis to a more contemporary population with a diagnosis after 1 January 2019. Preclinical and clinical evidence supports an immunologically cold TME in SCLC, which is frequently enriched with immune suppressive cells such as regulatory T cells, myeloid-derived suppressor cells, and TAMs. Moreover, most SCLC tumors exhibit an immune-desert tumor bed, which negatively affects the anti-PD-(L)1 blockade.^21^ In addition, the antigen presentation machinery is often defective in SCLC.^22^ For all these reasons, chemotherapy-induced antigen release may not be efficiently translated into improved cancer recognition by the immune system in SCLC, potentially limiting the chemoimmunotherapy synergistic effect. Finally, SCLC aggressiveness leads to the rapid acquisition of resistance mechanisms through lineage plasticity and epigenetic reprogramming, which are poorly regulated by the immune compartment.^23^

Consistently, our study showed no difference in rw-PFS—which is a better surrogate for treatment-specific effect than OS—between patients previously exposed to immunotherapy and those who were not. These findings argue against a clinically meaningful synergistic effect between chemotherapy and PD-1/PD-L1 blockade beyond the first line. Further prospective validation is warranted, as these results have important implications for treatment sequencing and for avoiding premature use of effective second-line options. Recently, the anti-DLL3 T cell engager tarlatamab received FDA approval in the second-line setting after phase III trial results demonstrating superiority over chemotherapy in relapsed SCLC patients.^24^ Understanding the potential clinical benefit of combining chemotherapy with other TME-targeting agents beyond PD-1/PD-L1 inhibitors remains an open and clinically relevant question, particularly with respect to patients’ quality of life.

Several limitations of the present study should be acknowledged. Patients who did not receive an immunotherapy-based strategy in the first-line setting had a longer follow-up compared with those who did, largely because many were diagnosed before immunotherapy became the standard of care. Although a minimum follow-up of 6 months from the initiation of second-line treatment was required to ensure PFS comparability, a potential overestimation of OS in the immunotherapy-pretreated group cannot be excluded. Additionally, subgroup analyses by treatment option were limited by relatively small sample sizes (∼50 patients per arm), reducing the robustness of conclusions regarding the specific impact of previous immunotherapy exposure on a given drug. Moreover, given the retrospective nature of the dataset, inaccuracies in date reporting may have contributed to the inability to classify ∼19% of second-line treatments into standard-of-care categories. Finally, the collected medical records did not allow us to account for potentially impactful variables such as supportive care, the type of imaging used for staging (particularly for brain assessment), and heterogeneity in patient follow-up.

Despite these limitations, this study represents the first large real-world analysis investigating the potential impact of prior immunotherapy exposure on second-line chemotherapy outcomes in SCLC. The large sample size enabled adjustment for imbalances in key prognostic factors, which is often an important limitation of retrospective studies.

In conclusion, this study showed no OS benefit from second-line treatment in immunotherapy-pretreated patients compared with those who received chemotherapy alone in the first-line setting. No difference in rw-PFS was observed, and no specific chemotherapy agent demonstrated differential efficacy according to the first-line regimen. These findings suggest limited or no synergistic activity between chemotherapy and immunotherapy beyond the first-line setting in SCLC.

## Declaration of AI and AI-Assisted technologies in the writing process

During the preparation of this work, the authors used Grammarly in order to improve grammar spelling and word flow. After using this tool/service, the authors reviewed and edited the content as needed and take full responsibility for the content of the published article.
