# Supplementary material for: Second-line chemotherapy efficacy is not affected by prior exposure to immunotherapy in extensive-disease small-cell lung cancer: results from the ESME French national real-world cohort
Source: ESMO Open. 2026 Aug 7;11(8):108324. doi: 10.1016/j.esmoop.2026.108324 (PMC13476521; doi:10.1016/j.esmoop.2026.108324)
Supplement: Supplementary file 1 [file mmc1.docx]

**Second Line Chemotherapy Efficacy is not affected by prior Exposure to Immunotherapy in extensive Small-Cell-Lung Cancer: results from the ESME French National Real World Cohort**

**Supplementary Materials**

**Supplementary Figure1:** Second-line Progression Free Survival Kaplan Mayer analysis according to the treatment option and upfront strategy

**Supplementary Figure 2 :** Second-line PFS Cox regression analysis by second line treatment option with (IPTW) and without propensity score

**Supplementary Figure 3:** Second-line OS Cox regression analysis by second line treatment option with (IPTW) and without propensity score

**Supplementary Table 1:** Comorbidities in the Study Population

**Supplementary Table 2:** Treatment choice in second line setting

**Supplementary Table 3:** Second-line PFS Cox univariate analysis for clinical characteristics

**Supplementary Figure 1:**

**Supplementary Figure 2:**

**Supplementary Figure 3:**

**Supplementary Table 1:**

|  | | | **Immunotherapy in L1** | |  |
| --- | --- | --- | --- | --- | --- |
| **Group** | **Variable** | **N** | **No**  N = 1,413 | **Yes**  N = 638 | **p-value** |
| **All patients** | **Medical history, n (%)** | 1,967 |  |  | 0.44^1^ |
|  | No |  | 608 (44.97) | 265 (43.09) |  |
|  | Yes |  | 744 (55.03) | 350 (56.91) |  |
|  | Unknown |  | 61 | 23 |  |
| **Only patients with medical history** | **Chronic obstructive pulmonary disease (copd), n (%)** | 1,094 |  |  | 0.12^1^ |
|  | No |  | 524 (70.43) | 230 (65.71) |  |
|  | Yes |  | 220 (29.57) | 120 (34.29) |  |
|  | Unknown |  | 0 | 0 |  |
|  | **Asbestosis, n (%)** | 1,094 |  |  | >0.99^2^ |
|  | No |  | 742 (99.73) | 349 (99.71) |  |
|  | Yes |  | 2 (0.27) | 1 (0.29) |  |
|  | Unknown |  | 0 | 0 |  |
|  | **Kidney failure, n (%)** | 1,094 |  |  | 0.001^1^ |
|  | No |  | 709 (95.30) | 347 (99.14) |  |
|  | Yes |  | 35 (4.70) | 3 (0.86) |  |
|  | Unknown |  | 0 | 0 |  |
|  | **Tuberculosis, n (%)** | 1,094 |  |  | 0.26^1^ |
|  | No |  | 715 (96.10) | 341 (97.43) |  |
|  | Yes |  | 29 (3.90) | 9 (2.57) |  |
|  | Unknown |  | 0 | 0 |  |
|  | **Heart failure, n (%)** | 1,094 |  |  | 0.16^1^ |
|  | No |  | 712 (95.70) | 341 (97.43) |  |
|  | Yes |  | 32 (4.30) | 9 (2.57) |  |
|  | Unknown |  | 0 | 0 |  |
|  | **Diabetes mellitus, n (%)** | 1,094 |  |  | 0.80^1^ |
|  | No |  | 526 (70.70) | 250 (71.43) |  |
|  | Yes |  | 218 (29.30) | 100 (28.57) |  |
|  | Unknown |  | 0 | 0 |  |
|  | **High blood pressure, n (%)** | 1,094 |  |  | 0.51^1^ |
|  | No |  | 215 (28.90) | 108 (30.86) |  |
|  | Yes |  | 529 (71.10) | 242 (69.14) |  |
|  | Unknown |  | 0 | 0 |  |
|  | **Psoriasis, n (%)** | 1,094 |  |  | 0.21^1^ |
|  | No |  | 733 (98.52) | 341 (97.43) |  |
|  | Yes |  | 11 (1.48) | 9 (2.57) |  |
|  | Unknown |  | 0 | 0 |  |
|  | **Rheumatoid arthritis, n (%)** | 1,094 |  |  | 0.59^1^ |
|  | No |  | 730 (98.12) | 345 (98.57) |  |
|  | Yes |  | 14 (1.88) | 5 (1.43) |  |
|  | Unknown |  | 0 | 0 |  |
|  | **Hemorrhagic rectocolitis, n (%)** | 1,094 |  |  | >0.99^2^ |
|  | No |  | 742 (99.73) | 349 (99.71) |  |
|  | Yes |  | 2 (0.27) | 1 (0.29) |  |
|  | Unknown |  | 0 | 0 |  |
|  | **Crohn’s disease, n (%)** | 1,094 |  |  | 0.60^2^ |
|  | No |  | 742 (99.73) | 348 (99.43) |  |
|  | Yes |  | 2 (0.27) | 2 (0.57) |  |
|  | Unknown |  | 0 | 0 |  |
| ^1^Pearson's Chi-squared test | | | | | |
| ^2^Fisher's exact test | | | | | |

**Supplementary Table 2:**

|  |  | **Immunotherapy in 1L** | |
| --- | --- | --- | --- |
| **Second-line regimen** | **Overall**  N = 2051 | **No**  N = 1413 | **Yes**  N = 638 |
|  |  |  |  |
| LURBINECTEDINE n (%) | 189 (9.2) | 50 (3.5) | 139 (22) |
| IRINOTECAN/TOPOTECAN n (%) | 492 (24) | 410 (29) | 82 (13) |
| PLATIN + ETOPOSIDE, n (%) | 632 (31) | 414 (29) | 218 (34) |
| PLATIN + TAXANES, n (%) | 159 (7.8) | 94 (6.7) | 65 (10) |
| CYCLOPHOSPHAMIDE, ADRIAMYCIN, VINCRISTINE (CAV), n (%) | 183 (8.9) | 132 (9.3) | 51 (8.0) |
| OTHER REGIMENS n(%) | 396 (19) | 313 (22) | 83 (13) |

**Supplementary Table 3:**

|  | | | **Univariate Cox regression** | |
| --- | --- | --- | --- | --- |
| **Characteristic** | **N** | **Event N** | **HR** **(95% CI)**^1^ | **p-value** |
| **Immunotherapy in L1** |  | 2,016 |  | 0.32 |
| No | 1,413 |  | — |  |
| Yes | 638 |  | 0.95 (0.87 to 1.05) |  |
| **Gender** |  | 2,016 |  | **0.002** |
| Female | 779 |  | — |  |
| Male | 1,272 |  | 1.15 (1.05 to 1.26) |  |
| **Age at the start of second-line treatment** |  | 2,016 |  | 0.60 |
| <65 | 1,014 |  | — |  |
| 65-75 | 777 |  | 1.03 (0.93 to 1.13) |  |
| >75 | 260 |  | 0.96 (0.83 to 1.10) |  |
| **Performance status at second-line** |  | 2,016 |  | **<0.001** |
| 0/1 | 843 |  | — |  |
| Missing | 712 |  | 1.09 (0.99 to 1.21) |  |
| 2+ | 496 |  | 1.82 (1.62 to 2.04) |  |
| **Smoking** |  | 1,932 |  | 0.53 |
| Current smoker | 918 |  | — |  |
| Former smoker | 989 |  | 1.01 (0.92 to 1.10) |  |
| Non smoker | 58 |  | 0.87 (0.66 to 1.13) |  |
| **Cumulative number of metastatic sites at start of second-line treatment** |  | 2,016 |  | **<0.001** |
| ≤ 3 | 1,558 |  | — |  |
| > 3 | 493 |  | 1.39 (1.25 to 1.54) |  |
| **Platine sensitivity in L1** |  | 2,016 |  | **<0.001** |
| No | 1,280 |  | — |  |
| Yes | 771 |  | 0.49 (0.45 to 0.54) |  |
| ^1^HR = Hazard Ratio, CI = Confidence Interval | | | | |
